# EchoMamba: A new Mamba model for fast and efficient hyperspectral image classification

**DOI:** 10.1371/journal.pone.0330678

**Published:** 2025-08-21

**Authors:** Yancong Zhang, Xiu Jin, Xiaodan Zhang, Yuting Wu, Lijing Tu

**Affiliations:** 1 College of Information and Artificial Intelligence, Anhui Agricultural University, Anhui, China; 2 Key Laboratory of Agricultural Sensors, Ministry of Agriculture and Rural Affairs, Anhui Agriculture University, Anhui, China; National Taiwan Ocean University, TAIWAN

## Abstract

The classification of hyperspectral images (HSI) is an important foundation in the field of remote sensing. Mamba architectures based on state space model (SSM) have shown great potential in the field of HSI processing due to their powerful long-range sequence modeling capabilities and the efficiency advantages of linear computing. Based on this theoretical basis, We propose a novel deep learning framework: long-sequence Mamba (EchoMamba), which combines the powerful long sequence processing capabilities of Long Short-Term Memory(LSTM) and Mamba to further explore the spectral dimension of HSI, and carry out more in-depth mining and learning of the spectral dimension of HSI. Compared with the previous HSI classification model, the experimental results show that EchoMamba can significantly reduce the training time cost of HSI and effectively improve the performance of the classification task.This study not only advances the current state of HSI classification but also provides a robust foundation for future research in spectral-spatial feature extraction and large-scale remote sensing applications.

## Introduction

HSI is a kind of image data containing rich spectral information, which can effectively detect the spectral information of ground objects from visible band to thermal infrared band through hundreds of spectral channels. With the nanoscale spectral resolution, the hyperspectral sensor can continuously perform spectral scanning and record the spectral reflectivity of each band, thus forming a continuous spectral curve, which reflects the spectral characteristics of the ground objects, which are closely related to the composition, structure, state and other factors of ground objects [1].Through in-depth analysis of these spectral curves, hyperspectral sensors can identify and distinguish different ground objects, so as to achieve accurate classification and feature extraction of land cover.

With its superior spectral resolution, HSI plays an important role in several fields. In the field of agriculture, by analyzing the spectral characteristics of crops at different growth stages, HSI can realize effective monitoring of crop growth, diseases, pests and nutritional status [2]. In terms of environmental monitoring, it can monitor water pollution, atmospheric composition, soil characteristics and other key environmental factors [3]. In geological exploration, HSI helps to identify mineral composition and provide support for the exploration of geological structures and mineral deposits [4]. In urban planning, it can be used for land cover classification and urban heat island effect analysis to support smart city development [5]. In addition, in the field of military reconnaissance, it also plays an important role, such as performing tasks such as target identification and camouflage detection [6].

Today, deep learning technology has made remarkable progress in dealing with HSI [7]. The success of deep learning in this domain can be attributed to its ability to automatically extract hierarchical and discriminative features from high-dimensional and complex data. Unlike traditional methods that often rely on handcrafted features and prior knowledge, Models such as convolutional neural networks (CNN) can learn spatial-spectral features directly from raw HSI data, capturing intricate patterns and relationships that are difficult to model manually. This is especially beneficial for HSI, where the data contains rich spectral information across hundreds of narrow bands, enabling the identification of subtle material differences and fine-grained classification. Additionally, Some deep learning models such as recurrent neural networks (RNN), Transformers, could further enhanced the capability to model sequential dependencies and spatial relationships in HSI data. The scalability and adaptability of deep learning models also make them suitable for various HSI applications [8].

CNN can effectively extract the spatial features of images through convolutional layer [9], which is very effective for HSI classification and target detection tasks. In addition, the multi-layer structure of CNN allows the model to learn hierarchically from low-level features to high-level features, which can better learn the rich spatial features of HSI in the training stage of the model [10]. However, traditional CNN models tend to pay too much attention to the spatial features and ignore the effective use of spectral information when processing HSI, thus limiting the model’s processing performance of HSI. In addition, due to the huge amount of HSI, it is also a great challenge to use CNN to train HSI.

Through its unique parameter-sharing mechanism, RNN can effectively reduce the number of parameters in the model [11], which can significantly reduce the time cost compared with CNN when processing HSI, thus effectively improving the operational efficiency of the model [12]. However, in terms of processing performance, the sequence data processing characteristics of RNN expose certain limitations in the face of complex data with the characteristics of high-dimensional such as HSI. Since RNN needs to process each element of the sequence in turn when processing data, it has serious shortcomings in spatial feature extraction. Given the rich spatial complexity in HSI, RNN struggle to capture some long-distance dependencies and local features in the image, which leads to certain limitations in its performance in HSI classification tasks.

As a relatively new large model, Transformer has shown excellent performance in the field of HSI processing in recent years. Through its self-attention mechanism, Transformer can effectively capture long-distance dependencies in images, thus realizing accurate understanding of the global context in HSI [13]. Different from RNN, Transformer can process all elements in the sequence in parallel, which further improves the speed of model training and reasoning on HSI. By incorporating positional encoding, Transformers preserve the position information of pixels in the image, which is very important for the spatial position analysis of HSI [14]. However, although the self-attention mechanism is very effective in HSI processing, the quadratic computational complexity of self-attention mechanisms necessitates a large amount of computing resources and memory when processing HSI with complex dimensions, which is even higher than that of CNN. In addition, due to the complexity of the internal mechanism of Transformer, its decision-making process often lacks transparency, which makes it extremely challenging in terms of model interpretation.

Mamba is a new deep learning framework inspired by the Structured State Space Model (SSM) [15], which is a model used to deal with long sequence efficiency problems, but such models have great limitations in terms of application scenarios, and their performance is poor when dealing with modes such as languages. Mamba improves on this by making the parameters of the SSM a function of the input, which allows the improved model to selectively propagate information along the sequence length dimension based on the tokens currently being processed. Although the improved SSM cannot use convolution to speed up data processing, Mamba has designed a hardware-friendly parallel algorithm that enables efficient processing of data in circular mode; In addition, because Mamba presents linear scaling O(n) with sequence length, it is able to efficiently process large data volumes. Benchmark experiments demonstrate that when performing the same task, compared with Transformer, Mamba can achieve a nearly 5 × speedup in inference speed while ensuring output quality.

Based on its strong adaptability to long sequence data [16], Mamba is able to effectively process HSI. The content-based reasoning ability of allows it to selectively perform propagation-or-forget operations based on the information in each band in the HSI, thus capturing spectral features more efficiently. In addition, Mamba’s advanced performance on multiple modes and good generalization ability enable it to effectively adapt to data types with complex dimensions such as HSI. In view of the huge amount of data in HSI, Mamba’s hardware-optimization parallel algorithm also enables it to effectively utilize computing resources and realize efficient processing of HSI [17]. However, Mamba’s ability to process data depends to a certain extent on the quality of the data itself. In the case of poor HSI data quality, the performance of the model may suffer and thus reduce the ability to process HSI. Therefore, before using Mamba to process HSI, HSI preprocessing becomes a significant challenge.

In order to further explore the potential of Mamba in HSI processing, a novel Long-sequence Mamba(EchoMamba) framework is proposed in this paper. On the basis of full reference and fusion of existing technologies, the framework adopts a series of efficient data preprocessing strategies, including feature selection and oversampling, to ensure the quality and reliability of HSI data. On this basis, EchoMamba cleverly combines short-term memory network (LSTM) with original Mamba to form a highly innovative deep learning model. This new framework not only inherits Mamba’s unique advantages in processing HSI, but also enhances the model’s processing capability of HSI spectral dimensions through LSTM, providing more powerful technical support for HSI classification, recognition and analysis.

## Materials & methods

### Hyperspectral images: basic research

As an advanced remote sensing image, HSI plays an important role in many fields because of its unique data characteristics and wide application prospects. The image reveals the rich spectral features of ground objects through capturing the reflection or emission information of objects in different spectral bands. The HSI is presented as a three-dimensional data cube in terms of character [18], where each pixel contains not only a single characteristic value, but a complete spectral curve. Such a data structure gives Although it means that HSI can provide detailed spectral information about ground objects, which greatly promotes the analysis and classification of material composition, its huge amount of data is difficult to deal with, which is an unavoidable problem.

In the field of deep learning, in order to effectively process and analyze HSI, researchers have used CNN, RNN, Transformer and other technologies to make many attempts. Hsi-CNN is a convolutional neural network framework used to improve the classification performance of HSI [19]. According to the characteristics of HSI data, the framework is designed to extract the spectrum-space features of target pixels and their neighborhoods, convert these features into one-dimensional feature maps through convolution operations, and stack them into two-dimensional matrices, which are finally input into the standard convolutional neural network as images for classification. Instead of using convolution to process HSI, Zhang et al proposed a local spatial sequence method, LSS [20], which they embedded into RNN to extract local and semantic information of HSI, thus achieving classification of HSI. Specifically, they extracted and fused low-level features, including texture and differential morphological contours, through the analysis of HSI to construct LSS features. Then they used these features as inputs, an RNN model was trained to optimize the system parameters of the network. At the end of the network, the high-level semantic features generated by the RNN are fed into the Softmax layer for final classification decisions.ConGCN is an HSI classification model based on graph convolution network and contrastive learning [21]. To solve the problem of insufficient supervision caused by the scarcity of HSI labeled samples, CONGCN integrates spectrum-space contrast supervision and adaptive graph structure optimization to improve the classification performance. In this model, a semi-supervised contrast learning mechanism is designed to implement feature consistency constraints on the nodes of the same object category or the enhanced view of the same node, and the implicit spectral discrimination information is mined from the limited markers. Secondly, the potential spatial relationships between pixels are captured by the graph-generation loss guidance model, and the topological relationships of adjacent similar nodes are converted into auxiliary supervisory signals. At the same time, combined with spectral similarity and spatial distance, the node connection weights of the graph are dynamically adjusted, and the adaptive enhanced graph structure is constructed to enhance the rationality of feature comparison. Finally, the combined multi-source supervised graph convolutional network can effectively learn discriminative features under the condition of a few labels, and achieve classification accuracy beyond the traditional GCN and comparison model on six typical data sets.

According to the theoretical results of the researchers, we can conclude that CNN and RNN have their own advantages in the field of HSI. With its excellent ability of convolutional feature extraction, CNN are powerful in dealing with the spatial dimension of HSI, and can carry out feature learning efficiently. However, when faced with the spectral dimension with long sequence features, the processing ability of CNN is slightly insufficient. On the contrary, relying on its unique cyclic characteristics, RNN have significant advantages in deep mining the spectral dimension of HSI, but their performance is relatively limited when dealing with more complex spatial information containing two dimensions.

Considering that it is difficult to fully excavate the multi-dimensional deep information when using CNN, GCN or RNN alone to process HSI, He et al proposed an innovative Space-Spectral Transformer (SST) classification framework based on the theory of Transformer’s HSI processing. This framework cleverly combines CNN to extract spatial features of images, and captures long-distance spectral sequence relationships through an improved Transformer structure. Based on this, multi-layer perceptrons are utilized for the classification task. Compared with the previous methods, the SST framework can handle HSI more comprehensively, and demonstrates significant improvements. However, due to the complexity of its model structure, SST has a O(n^2^) computational complexity for the HSI training phase, which indicates that there are still some challenges and limitations when using Transformer for HSI processing.

To sum up, existing approaches to HSI classification, including CNN, RNN and GCN-based methods, continue to face performance limitations. Although Transformer models show promising results in this domain, their application is constrained by substantial computational requirements.

### Mamba: reference model

Mamba has shown excellent applicability in HSI training, mainly due to its strong feature extraction and timing modeling capabilities [22]. HSI has high dimensionality, high correlation and rich spectral information, which makes traditional models often face problems such as high computational complexity and difficulty in feature extraction when processing such data. Mamba, through its core component SSM, is able to effectively capture continuous spectral information in HSI, thus achieving excellent performance in tasks such as classification, identification and anomaly detection.

**Fig 1:**
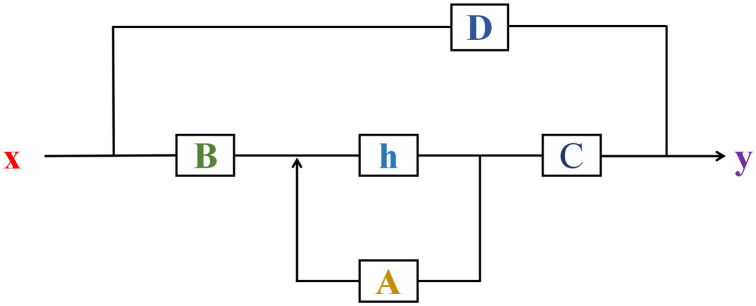
State Space Model(SSM) framework.


Fig 1 shows the basic framework of SSM. SSM is constructed from a continuous-time system [23], which maps the input sequence x(t) to the corresponding output signal y(t) through an potential state h(t).

$$\begin{array}{c} {\text{h}}^{\prime }(\text{t})=\text{Ah}(\text{t})+\text{Bx}(\text{t}) \end{array}$$ (1)

y(t)=Ch(t)+Dx(t) (2)

Formula (1) describes the dynamic evolution process of h(t) with time t. Where $\begin{array}{c} {\text{h}}^{\prime }(\text{t}) \end{array}$ represents a hidden intermediate state in the model from x(t) to y(t). In this model, the state transition matrix $\text{A}\in {\text{R}}^{\text{N}*\text{N}}$, which precisely characterizes the state transition mechanism of h(t) from one moment to the next on the timeline. In other words, A determines how state h(t) transitions between different points in time, which allows the model to predict where the state will go in the future. B $\in {\text{R}}^{\text{N}*\text{M}}$,which is a control matrix that reveals the extent to which x(t) contributes to changes in the h(t). Specifically, the B describes how the x(t) can influence the dynamics of h(t) through a complex series of interactions. This formula shows that the change in h(t) is a result of the interaction between the h(t) and the x(t). This interaction is not only continuous in time, but also shows the response ability of the system to the external stimulus.

Formula (2) illustrates the process by which h(t) and x(t) generate y(t). As the observation matrix $\text{C}\in {\text{R}}^{\text{O}*\text{N}}$, it extracts the information related to the output in h(t), transforming it into a part of y(t). D$\in {\text{R}}^{\text{O}*\text{M}}$, which is a direct transfer matrix that describes the process by which x(t) is passed directly to y(t) without going through h(t), which can be seen as a residual connection that allows the input signal to have a direct effect on the output. By combining the linear transformation of C to h(t) with the direct transfer of D to x(t), Formula (2) obtained the final output y(t).

However, SSM faces a significant efficiency bottleneck when dealing with long sequences and large volumes of data. This is because the memory and computation time required by the SSM model show a linear increase trend as the length of the sequence increases. This characteristic limits the performance of SSM when dealing with some discrete signal processing tasks. In practical applications, this bottleneck may lead to slow processing speed, excessive resource consumption, and even the inability to process ultra-long sequences or large-scale data sets, thus affecting the applicable scope and popularization value of the model

To solve this problem, the researchers use zero-order preservation technique to discretize the system parameters in SSM. Zero-order hold technique is a discretization method commonly used in digital signal processing. Based on this technique, the re-parameterization process of system matrix and time scale parameters is as follows:


$$\overset{―}{\text{A}}=\text{exp}(\Delta \text{A})$$ (3)

$$\overset{―}{\text{B}}={\left(\Delta \text{A}\right)}^{-\text{1}}(\overset{―}{\text{A}}-\text{I})(\Delta \text{B})$$ (4)

$$\approx {\left(\Delta \text{A}\right)}^{-\text{1}}(\Delta \text{A})(\Delta \text{B})$$

=ΔB

Formula 3 achieves discretization of the A, where ΔA is the product of A and the sampling time interval Δ. In discrete-time system, the state transition matrix $\overset{―}{\text{A}}$ is obtained by performing an exponential operation on A, where the exponential operation of A can be computed by summing the Taylor series expansions of the ΔA, As shown in Formula 5.


$$\text{exp}(\Delta \text{A})=\text{I}+\Delta \text{A}+\frac{{\left(\Delta \text{A}\right)}^{\text{2}}}{\text{2}!}+\frac{{\left(\Delta \text{A}\right)}^{\text{3}}}{\text{3}!}+...+\frac{{\left(\Delta \text{A}\right)}^{\text{n}}}{\text{n}!}$$ (5)

In Formula 4, ΔB is the product of B and Δ, this formula is actually an approximation calculation that uses a first-order Taylor series to approximate the expansion $\overset{―}{\text{B}}$, which is finally obtained$\;\text{result}\;\overset{―}{\text{B}}=\Delta \text{B}$.

After discretizing the parametric matrix, the discrete SSM can be formulated as the following cyclic representation:


$${\text{h}}_{\text{k}}=\overset{―}{\text{A}}{\text{h}}_{\text{k}-\text{1}}+\overset{―}{\text{B}}{\text{x}}_{\text{k}}$$ (6)

$${\text{y}}_{\text{k}}=\text{C}{\text{h}}_{\text{k}}$$ (7)

Formulas 6 and 7 together define the discretized SSM, which can be used for dynamic systems in simulation and control theory, as well as for processing sequence data such as language, audio, and images. By expanding the state-space model into a recursive relationship, it can be processed in a computational framework similar to RNN, and training efficiency can be improved by GPU acceleration.


$$\text{y}=\text{x}*\overset{―}{\text{k}}$$ (8)

In order to better adapt to the efficient parallel computing characteristics of GPU, Formula 8 further optimizes this recurrence relationship. The original recurrence relationship is expanded and transformed into the form of global convolution, where is the convolution kernel $\overset{―}{\text{k}}$, which is composed of a series of matrix products of discretized SSM, the specific form is as Formula 9:


$$\overset{―}{\text{k}}=(\text{C}\overset{―}{\text{B}},\text{C}\overset{―}{\text{A}}\overset{―}{\text{B}}\text{,}...\text{M},\text{C}{\overset{―}{\text{A}}}^{\text{L}-\text{1}}\overset{―}{\text{B}})$$ (9)

The conversion of Formula 9 not only improves the parallelism of the computation, but also enables the GPU to make more efficient use of its powerful computing resources when processing large-scale data. In this way, discretizing SSM significantly improves the algorithm’s speed, providing a solid foundation for processing certain tasks of discretizing signal data.

In addition to the limitation of linear problems, SSM also has the assumption of linear time invariance (LTI). This means that the model parameters remain constant over each time step and do not have the property of changing over time. This assumption makes it impossible for SSM to update parameters in time when processing data with time-varying dynamic behavior, thus limiting the expressiveness of the model.

Selective SSM (S6) is a new type of system state model, which breaks the constraints of LTI by parameterizing parameter (Δ,B,C) as a function of input x, so that SSM can obtain more powerful selection ability and can focus on important content while ignoring unimportant information. Based on the core structure of S6, the researchers propose the Mamba framework, which is a simplified neural network architecture that includes linear layers, convolutional layers, residual connections, nonlinear transformations and, most importantly, the S6 kernel. In order to ensure the efficient implementation of Mamba, the researchers also propose an optimization strategy for the hardware, so that Mamba can dynamically adjust parameters according to different inputs, and better process information.

In the process of in-depth study on the HSI processing field combined with Mamba theory, researchers have carried out a variety of improvements and optimization measures to the Mamba framework. Based on the theoretical basis of Mamba, He et al. proposed a 3D-Spectral-Spatial Mamba (3DSS-Mamba) framework [24]. 3DSS-Mamba focuses on the extraction of spectrum-spatial information in three-dimensional space. The framework transforms the HSI cube into a set of 3D Spectral-Spatial Selective Scanning (3DSS) by designing a spectral-spatial label generation (SSTG) module, and introduces a 3D-spectral Spatial Selective Scanning (3DSS) mechanism. This mechanism enables pixel-wise selective scanning of 3D hyperspectral markers in spectral and spatial dimensions to overcome the limitations of traditional Mamba in dealing with high-dimensional scenes. In addition, He et al. constructed 5 scanning paths to study the influence of dimensional priority, and combined the 3DSS scanning mechanism with traditional mapping operations to form a 3D spectrum-space Mamba block (3DMB), thus realizing the extraction of global spectrum-space semantic representation and improving computational efficiency.

**Fig 2:**
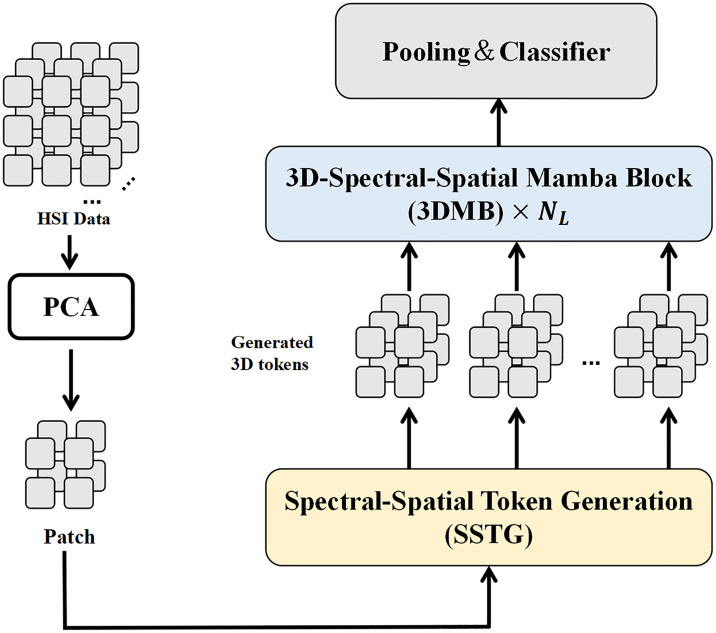
Architecture of 3DSS-Mamba framework.

Fig 2 shows the basic process framework of 3DSS-Mamba. 3DSS-Mamba firstly reduces the dimensionality of the input HSI data through principal component analysis (PCA) to extract the key spectral information, and then converts the processed data into a set of 3D spectral spatial labels through the Spectral-Spatial Token Generation (SSTG) module. These tags are then input into 3D-Spectral Spatial Mamba blocks (3DMB). Within each 3DMB block, specific pooling and classification operations are performed for further feature extraction and classification prediction of the tags. Finally, the output of all 3DMB blocks is summarized. The final HSI classification result is obtained through the pooling layer and classifier, and the whole process effectively combines spectral and spatial information to achieve efficient HSI classification. As an advanced HSI classifier, 3DSS-Mamba reflects the trend of sequential processing in HSI classification. Researchers usually treat the spectral dimension as a potential continuous sequence for processing. This trend demonstrates that the spectral bands of HSI have long sequence characteristics similar to time series.

According to this improved method, it can be seen that the trend and direction of the improvement of Mamba by researchers mainly focus on the deep fusion of spatial and spectral information to improve the accuracy of HSI classification, and pay attention to the improvement of the computational efficiency of variant models to ensure that the performance is enhanced without sacrificing the computing speed [25]. These improvements also include the innovation of the model structure to better capture the local and global features of the image, enhance the robustness of the model, and enable it to adapt to different data variations [26]. In addition, researchers tend to optimize the process of feature extraction and representation learning through more efficient feature coding to enhance model interpretability [27]. These improved strategies further promote the performance of the model in practical application, bring a new research perspective and methodology to the HSI processing field, and also provide a wealth of inspiration and reference for the subsequent research work of this paper. Based on the theoretical foundation of Mamba model in HSI field, we construct a new variant model EchoMamba. In the following sections, we will introduce this model in detail.

### EchoMamba: preliminaries

In order to further improve Mamba’s performance in processing HSI data, we deeply optimize and innovate its processing techniques in spatial dimension and spectral dimension on the basis of inheriting the traditional Mamba theory. This improvement aims to comprehensively improve the application effect of Mamba in the field of HSI analysis.

For the spatial dimension of HSI data, Since Mamba itself is more suitable for processing continuous data types, if only a single Mamba is used to process the spatial dimensions of HSI, it is necessary to first mark the positions of each data in the spatial dimensions of HSI, and then flatten the overall three-dimensional dimension to treat it as data of a continuous dimension. Each position (x, y) that was previously marked is processed in turn by Mamba.


$${\text{h}}_{\text{x},\text{y}}(\text{t})=\text{A}{\text{h}}_{\text{x},\text{y}}(\text{t}-1)+\text{Bu}(\text{t})+\omega (\text{t})$$ (10)

$${\text{y}}_{\text{x},\text{y}}(\text{t})=\text{C}{\text{h}}_{\text{x},\text{y}}(t)+Du(t)+(t)\omega (t)$$ (11)

Formula 10,11 is Mamba’s improved process for HSI spatial dimensions. In the state-space model, ${\text{h}}_{\text{x},\text{y}}(\text{t})$ represents the hidden state at the spatial position (x,y) at the time step t. The state is updated by combining the state transition matrix A with the state at the previous moment and the control input matrix B with the external input u(t), and adding process noise ω(t). ${\text{y}}_{\text{x},\text{y}}(\text{t})$ represents the observed data position (x,y) at the time step t, which is associated with the hidden state and external input through the observation matrix C and the direct transfer matrix D, and is affected by the observation noise ω(t). For the spatial features of the global image, Mamba’s processing process is shown in Formula 12,13.


$${\text{H}}_{\text{x},\text{y}}(\text{t})=\text{A}{\text{H}}_{\text{x},\text{y}}(t-1)+BU(t)+\omega (t)$$ (12)

$${\text{Y}}_{\text{x},\text{y}}(\text{t})=\text{C}{\text{H}}_{\text{x},\text{y}}(t)+DU(t)+\omega (t)$$ (13)

Where ${\text{H}}_{\text{x},\text{y}}(\text{t})$ and ${\text{Y}}_{\text{x},\text{y}}(\text{t})$ are respectively the potential states and outputs of all pixel spatial features in the time step t.

For the spectral dimension, we treat the multiple bands of HSI pixels as a continuous time series. Based on the previous theoretical basis, we improve it and construct a formula to capture the long-term dependence on the spectral dimension.


$${\text{h}}_{\text{b}}(\text{t})=\text{A}{\text{h}}_{\text{b}}(\text{t}-\text{1})+\text{B}{\text{x}}_{\text{b}}(\text{t})$$ (14)

$${\text{y}}_{\text{b}}(\text{t})=\text{C}{\text{h}}_{\text{b}}(\text{t})+\text{D}{\text{x}}_{\text{b}}(\text{t})$$ (15)

Formula 14 describes the recurrence process of the potential state of the spectrum. At time step t, the potential state ${\text{h}}_{\text{b}}(\text{t})$ of spectral band b is calculated from the potential state of the previous time step through the A and combined with the input data ${\text{x}}_{\text{b}}(\text{t})$ transformed by the B.In Formula 15, we transform the potential state through C, and introduce D to directly influence the output observation of spectral band b under time step t. Finally, the two results are added together to get the final output prediction value ${\text{y}}_{\text{b}}(\text{t})$.

In order to apply the Mamba layer to all spectral dimensions of HSI, we apply the above recurrence relationship to each pixel of the image (i,j) on the spectral band.


$${\text{h}}_{\text{b}}(\text{i},\text{j},\text{t})=\text{A}{\text{h}}_{\text{b}}(\text{i},\text{j},\text{t}-\text{1})+\text{B}{\text{x}}_{\text{b}}(\text{i},\text{j},\text{t})$$ (16)

$${\text{y}}_{\text{b}}(\text{i},\text{j},\text{t})=\text{C}{\text{h}}_{\text{b}}(\text{i},\text{j},\text{t})+\text{D}{\text{x}}_{\text{b}}(\text{i},\text{j},\text{t})$$ (17)

For the entire image, this process can then be expressed as:


$${\text{H}}_{\text{b}}(\text{t})=\text{A}{\text{H}}_{\text{b}}(\text{t}-\text{1})+\text{B}{\text{X}}_{\text{b}}(\text{t})$$ (18)

$${\text{Y}}_{\text{b}}(\text{t})=\text{C}{\text{H}}_{\text{b}}(\text{t})+\text{D}{\text{X}}_{\text{b}}(\text{t})$$ (19)

Where ${\text{H}}_{\text{b}}(\text{t})$ and ${\text{Y}}_{\text{b}}(\text{t})$ are respectively the potential states and outputs of spectral features of all pixels in the time step t.


$$\text{F}(\text{t})=\text{concrete}({\text{Y}}_{\text{x},\text{y}}(\text{t}),{\text{Y}}_{\text{b}}(\text{t}))$$ (20)

After using Mamba to process the spatial and spectral dimensions of HSI respectively, as shown in Formula 20, the features of these two dimensions are integrated and analyzed, and finally the complete processing process of HSI data is realized by Mamba.

In view of some limitations of Mamba model in processing HSI, especially in the face of poor data quality, its performance may be affected. We decided to optimize the model and improve its performance by combining other advanced deep learning neural network layers with the Mamba model. The core idea of this strategy is to combine the strengths of different network layers to form complementary advantages, so as to enhance the processing ability of the model for HSI data.

In the process of this exploration, Our goal is to further expand the application range of Mamba model in the HSI processing field and improve its performance in practical applications through this innovative fusion strategy on the basis of retaining the original advantages of Mamba model.

RNN is a deep learning model designed specifically for processing sequence data, and its core advantage is its ability to capture time series features in the data [28]. By introducing a cyclic structure, this network forms a unique “memory” mechanism, which enables the network to not only consider the current input information when processing sequence data, but also remember the previous information. This property gives RNNS a significant advantage when dealing with scenarios such as text, speech, time series data, etc.

Although RNN provide a powerful framework for processing sequence data, this architecture has encountered many limitations in some practical applications. For example, in the process of processing long sequence data, it is difficult for RNN to learn the long distance dependency during training, because in the process of back propagation, the gradient tends to decrease exponentially with the increase of time step, resulting in the network failing to capture the information at a long distance in the sequence and eventually causing the problem of gradient disappearance [29]. In addition, in contrast to the disappearance of gradient, the gradient of RNN will become very large when the model learning rate is set too high, the network depth is too deep and so on, which makes the network weight need to be updated on a large scale, and finally leads to the entire learning process of the model is extremely unstable.

A Long Short-Term Memory (LSTM) is a variant of an RNN in deep learning that is designed to solve the problems of gradient disappearance and gradient explosion that standard RNNS encounter when processing long sequence data [30]. At the heart of LSTM is its unique memory unit, which is capable of transmitting state information over a long period of time in the network, a property that is carefully regulated through three gate structures: forget gates, input gates, and output gates.the forget gate determines what information should be discarded from the memory unit, a process that prevents the accumulation of irrelevant information; The input gate is responsible for updating the state of the memory unit, by combining the old state and the new input information to decide what data needs to be stored; Finally, the output gate controls how the contents of the memory unit affect the next hidden state and output, ensuring that relevant information is passed on to the next layer of the network in a timely manner. This structure makes LSTM excellent when dealing with sequence data with long distance dependencies, such as time series analysis, language models, speech recognition and other fields.

LSTM excels at mining deep dependencies in time series data with its superior ability. Although this may seem to have no direct relation to the processing of hyperspectral image data, previous studies have shown that the spectral sequence of hyperspectral image data contains rich and complex internal connections, which makes hyperspectral image data, to some extent, exhibit a high similarity to time series data. In summary, we try to combine LSTM and Mamba model on the basis of a series of data preprocessing strategies, and construct a new variant model EchoMamba.

The core feature of HSI data lies in the rich spectral dimension information contained in each pixel. Although there are spatial correlations among HSI pixels, and this spatial information is equally important for tasks such as scene understanding, image segmentation and object detection, this characteristic is not the most distinctive advantage of HSI. Therefore, in the process of using EchoMamba to process HSI data, we intentionally downplay the model’s dependence on the spatial dimension of the image, and instead give full play to the advantages of LSTM and Mamba, regard the spectral sequence as a special time series, and deeply explore the rich information of the spectral dimension. To enable more accurate interpretation and analysis of HSI data by the model. In next section, we will take a more detailed and in-depth look at the architecture of EchoMamba.

### EchoMamba: strategy and structure

**Fig 3:**
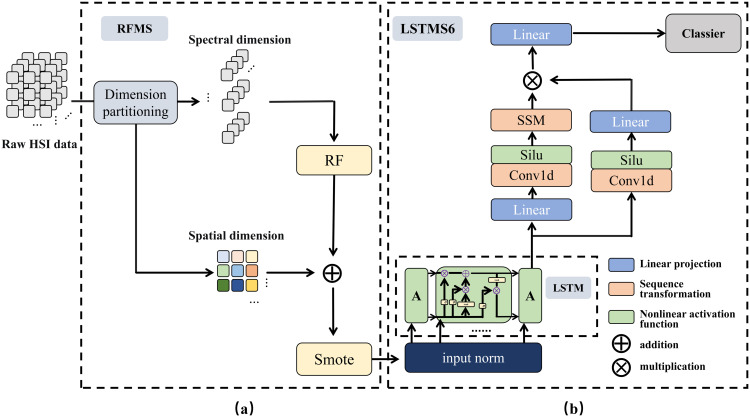
Architecture of the EchoMamba framework.

EchoMamba is an innovative variant that improves on the traditional Mamba model, building an entirely new model framework. As shown in Fig 3, we was structurally designed to improve the performance and efficiency of the model. The framework mainly includes two parts: the data preprocessing strategy Random Forest Smote(RFMS) and the variant model Long Short-Term Memory Mamba S6(LSTMS6).

Next, this article will delve deeper into these two core components of EchoMamba. We will elaborate on their functions, features, and performance in practical applications one by one, enabling comprehensive understanding of component functionalities and their important role in the overall architecture.

### EchoMamba strategy of data preprocessing

In spatial dimension, each pixel in the HSI represents a smaller area of the actual ground, which allows researchers to observe more detailed surface features in the image; In the spectral dimension, HSI usually contains tens to hundreds of continuous bands, the band width is usually between a few nanometers to tens of nanometers, covering the spectral range from visible light to near-infrared and even short-wave infrared, so that HSI can capture the subtle differences between ground objects in the spectral dimension. To sum up, HSI has extremely high resolution in both spatial dimension and spectral dimension.

However, although the unique data characteristics of HSI provide researchers with rich information, the complex dimension and huge amount of data also make its structure extremely complex. Therefore, RFMS adopts the techniques of feature selection and oversampling to process the different data features of HSI respectively.

While maintaining the spatial dimension of the original HSI data, we use random forest (RF) as our main method of feature extraction to extract the spectral dimension of the data. RF is an ensemble tree-based machine learning algorithm, which is often used for classification and regression tasks [31]. RF shows excellent performance in processing spectral dimension feature extraction of HSI.

The uneven distribution of samples is a common and challenging problem in HSI data analysis. There may be significant differences in the number of samples of different types of ground objects, and such unbalanced data distribution will adversely affect the training and performance of the model [32]. The evaluation index of the model may be too optimistic due to the domination of the majority class, and can not truly reflect its performance on the minority class, resulting in distortion of accuracy. In addition, the model relies too much on the characteristics of the majority class, which may lead to the lack of generalization ability in the face of minority class samples.

Synthetic Minority Oversampling Technique(SMOTE) has been applied to solve the problem of uneven sample distribution in HSI data effectively. The core idea of this algorithm is to create a batch of new synthetic samples with high authenticity by accurate interpolation between a few class samples of HSI data [33]. The aim is to significantly increase the number of samples in a few classes to balance the distribution of samples between the various classes and to avoid bias of the model against some classes during training.

Through the comprehensive application of a series of traditional methods, RFMS significantly improves the generalization ability of the model in HSI, further improves the accuracy of the prediction, and provides strong support for the effective analysis and application of HSI.

### EchoMamba structure of LSTM and Mamba

**Fig 4:**
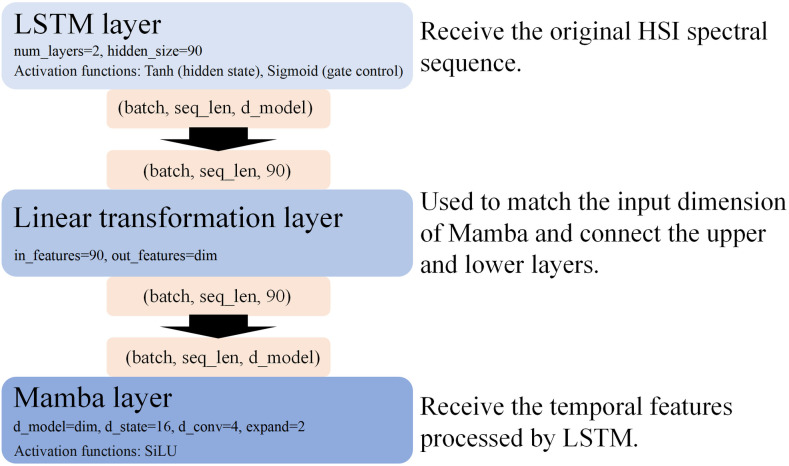
Details of the connection of the LSTM component.

LSTMS6 is the core architecture of the EchoMamba system, and its design is inspired by the theoretical foundation discussed earlier. On the basis of fully retaining the original structural advantages of the LSTM neural network layer and the S6 model, We cleverly spliced the two modules together, specifically, LSTM as a model block directly spliced with the S6 model. The S6 module, as the global context encoder of the spectral sequence, can extract the key spectral features and suppress the noise in the whole band range through its selective state space dynamic optimization mechanism. On this basis, LSTM layer, as a local temporal decoder, conducts a secondary modeling of the hidden state of S6 output through the coordination of the gate control unit: The forgetting gate dynamically adjusts the memory intensity of global features according to the current context, effectively eliminating the residual influence of environmental noise such as atmospheric interference; The input gate enhances the local temporal pattern with class discrimination through nonlinear transformation, thus forming a “global-local” two-granularity feature representation system. This cascading structure not only inherits the linear computational complexity advantage of S6 model for long sequence modeling, but also solves the limitation of traditional state space model in local nonlinear dynamic modeling through the temporal memory characteristic of LSTM. LSTMS6 specifically consists of three main components: the LSTM layer, the linear transformation layer, and the Mamba layer. The specific parameters and connection methods of each layer are shown in Fig 4. Algorithm 1 illustrates the specific implementation process of LSTMS6.

Algorithm 1: LSTMS6

1. **Input**: The number of samples in a batch: **batch_size**, The time step length of the input sequence: **seq_length**, Dimension of input features: **feature_dim**.

2. **Output**: **batch_size**, Dimension of output features: **output_dim**.

3. **For** each epoch ∈(1, 200), **do**

4. **inputs**(batch_size, feature_dim), **labels**(batch_size)←**train_loader**

5. **inputs**(batch_size, 1, feature_dim)←**inputs**.unsqueeze(1)

6. # Forward Propagation of LSTM Layer

7. **outputs_lstm**, (hn, cn) ← **LSTM**(inputs, (h0, c0))

8. # Linear Transformation for Dimension Adjustment

9. **outputs_linear** ← **Linear**(outputs_lstm)

10. # Mamba Layer Forward Propagation

11. **outputs_mamba** ← **Mamba**(outputs_linear)

12. **outputs** ← **outputs_mamba.squeeze**(1)

13. # Calculate Loss

14. **loss** ← **CrossEntropyLoss**(outputs, labels)

15. # Backpropagation (Gradient Calculation)

16. **d_loss** ← **loss**.backward()

17. # Mamba Layer Backpropagation

**18.** ∇**outputs_mamba** ← **gradients_from_loss**

19. # Linear Layer Backpropagation

20. ∇**outputs_linear** ← **Mamba**.backward(∇**outputs_mamba**)

21. # Backpropagation in LSTM Layer

22. ∇**outputs_lstm** ← **Linear**.backward(∇**outputs_linear**)

23. ∇**inputs**, (∇hn, ∇cn) ← **LSTM**.backward(∇**outputs_lstm**, (hn, cn))

24. # Parameter Update

25. **optimizer**.step()

 - **LSTM weights** (W_ii, W_if, W_ig, W_io)

 - **LSTM bias** (b_i, b_f, b_g, b_o)

 - **Linear layer weights** (W_linear)

 - **Linear layer bias** (b_linear)

 - **Mamba internal parameters**

However, in this fusion process, how to establish the hierarchical relationship between LSTM and Mamba has become a big challenge for us.

In order to solve this problem, we have carried out in-depth research and exploration. Through observation, we find that placing LSTM in front of S6 model can take advantage of LSTM’s advantages in processing abstract features, so that it can learn on higher-level features extracted and transformed by S6, which not only improves the level of time series understanding, but also optimizes the information flow, thus reducing the number of parameters in LSTM layer and improving the computational efficiency. At the same time, this structure helps to reduce the gradient disappearance problem, and enables LSTM to more directly propagate the prediction error to the S6 layer, which helps the S6 layer to better adjust the feature extraction strategy. In addition, for the domain-specific adaptability of HSI, LSTM can perform time series analysis more efficiently before S6, while the modular design also makes the model more flexible and easy to adjust according to different data sets and task requirements. As a result, the LSTM could theoretically achieve better results in front of the S6 layer. On the premise of ensuring the full play of the two characteristics, we designed a new structural layout, so that LSTM and S6 can coordinate and complement each other.

LSTMS6 significantly improves the performance of processing HSI time series relationship, feature extraction, computational efficiency and generalization ability through its innovative integration of structures. Table 1 illustrates the theoretical differences between EchoMamba and the various Mamba variants mentioned earlier in terms of architecture and functionality. The model utilizes the unique gating mechanism of the LSTM layer to effectively capture the long-term dependency relationship in image data and deeply understand the timing features. At the same time, combined with Mamba model’s expertise in feature extraction and transformation, it provides high-quality learning materials for the LSTM layer. So that the model can more effectively extract key features and perform efficient data transformation when dealing with complex HSI. In addition, by optimizing the hierarchical relationship, the LSTMS6 model effectively alleviates the problem of gradient disappearance, improves training efficiency and model performance, and thus shows higher accuracy in identifying and classifying HSI. The structural innovation also enables more efficient use of computing resources, reduced training time, and demonstrated advantages during deployment and application. At the same time, the model shows stronger generalization ability when facing HSI with different scenarios and conditions, achieving end-to-end learning from raw data to final prediction, simplifying the data processing process and improving the overall learning efficiency.

**Table 1:** Theoretical comparison of the Mamba variant model.

Framework	Function	Advantage
CNN+Mamba	Spatial-spectral separation modeling	Strong ability to capture spatial features
GCN+Mamba	Non-Euclidean relationship modeling	Skilled in long-distance spatial dependency and non-local feature interaction
Transformer+Mamba	Attention-enhanced sequence modeling	Stronger theoretical modeling ability
3DSS-Mamba	Empty spectrum combined three-dimensional modeling	Reasonable utilization of spatial and spectral information
EchoMamba	Dual exploration of spectral dimension depth	Comprehensive and detailed extraction of spectral characteristics

EchoMamba not only innovates in data processing and model structure design, but also provides a new way of thinking for HSI analysis and processing. In the next section, we will use the core component of EchoMamba, namely LSTMS6, to compare the performance of several other combined models, so as to prove the feasibility of this model.

## Results

### Dataset

Six internationally recognized HSI datasets were selected for model performance evaluation: Augsburg, Salinas, Pavia Centre scene, Pavia University, Indian Pines, Houston 2013. The following are detailed descriptions of each dataset:

Augsburg Dataset: This dataset covers part of the city of Augsburg, Germany, and was collected using a hyperspectral imaging spectrometer. It consists of 180 wavebands with image sizes of 332 * 485 pixels, divided into seven feature categories. The spatial resolution is 1 meter, which allows the features to be displayed in fine detail. In the entire data set, there are 161020 pixels, of which 78294 are ground objects and the rest are background pixels.

Salinas Dataset: This dataset originated in the Salinas Valley, California, USA, and was captured by the AVIRIS sensor. The dataset contains 224 bands with image sizes of 512 * 217 pixels divided into 16 feature categories. The spatial resolution is 3.7 meters, which ensures the identification and classification of the features. Of the 111104 pixels, 54129 are ground objects and the rest are background pixels.

Pavia Centre scene dataset: This dataset was taken over the city of Pavia, Italy, also by the AVIRIS sensor. The dataset consists of 102 wavebands with images of 1096 * 715 pixels grouped into nine feature categories. The spatial resolution is 1.3 meters, which provides high accuracy for the recognition of ground objects. There are 783640 pixels in the data set, of which 7456 are ground objects and the rest are background pixels.

Pavia University dataset: This dataset is part of the HSI acquired by the AVIRIS sensor in Pavia, Italy. It contains 103 spectral bands with an image size of 610 * 340 divided into nine different feature categories. The dataset has a spatial resolution of about 1.3 meters and a total of 207400 pixels, of which 42776 pixels are labeled as ground objects and the rest are background pixels.

Indian Pines Dataset: This dataset was acquired by an AVIRIS sensor in Indiana, USA, over the Indian Pines region. It contains 220 spectral bands with image sizes of 145 * 145 and covers 16 different feature categories. The spatial resolution is about 20 meters, and the entire dataset has a total of 21025 pixels, of which only 10249 pixels are in the feature category, and the rest are background pixels.

Houston 2013 dataset: It was acquired by ITRES CASI-1500 in and around the University of Houston campus. It contains 144 spectral bands, with an image size of 349 * 1905, grouped into 15 different feature categories. The data set has a spatial resolution of about 2.5 meters and a total of 664845 pixels, of which only 15029 pixels are labeled as ground objects and the rest are background pixels.

Table 2 shows the category distribution and quantity of each data set.

**Table 2:** Specific data sets and categories.

Class	Ausburg	Salinas	Pavia Centre scene	Pavia University	Indian Pines	Houston 2013
1	13507	2009	824	6631	46	1251
2	30329	3726	820	18649	1428	1254
3	3851	1976	816	2099	830	697
4	26857	1394	808	3064	237	1244
5	575	2678	808	1345	483	1242
6	1645	3959	1260	5029	730	325
7	1530	3579	476	1330	28	1268
8		11271	824	3682	478	1244
9		6203	820	947	20	1252
10		3278			972	1227
11		1068			2455	1235
12		1927			593	1233
13		916			205	469
14		1070			1265	428
15		7268			386	660
16		1807			93	
Total	78294	54129	7456	42776	10249	15029

### Basic experiment

#### Implementation details.

To facilitate reproducibility and fair comparison, all experiments were conducted using identical hardware and training configurations. The specific as detailed below:

Hardware Specifications
CPU: 11th Gen Intel® Core™ i7-11800H (8 cores, 16 threads @ 2.30 GHz)
GPU: NVIDIA GeForce RTX 3060 Laptop GPU (6GB GDDR6 VRAM)
RAM: 16GB DDR4 (2 × 8GB @ 3200MHz)
Storage: NVMe SSD (*employed to minimize I/O bottlenecks during data loading*)
Training Configuration
Batch size: 64
Epochs: 200
Optimizer: AdamW (learning rate = 0.001, weight decay = 1 × 10⁻⁴)
Details of EchoMamba’s model parameters
EchoMamba (LSTM+Mamba) Parameters: 201,561 (approximately 0.20 million)
EchoMamba Model File Size: 0.77 MB (FP32 precision)

This standardized setup ensures that performance differences observed between the core EchoMamba model and the benchmark models (Mamba, CNN+Mamba, GCN+Mamba, Transformer, CNN+Transformer, GCN+Transformer, LSTM+Transformer) are attributable to their architectural differences rather than variations in training conditions or hardware utilization.

### Validation set performance comparison

Based on Mamba and Transformer infrastructures, we build two sets of models by integrating CNN、GCN and LSTM layers respectively. Including eight models: Mamba, CNN+Mamba, GCN+Mamba, LSTM+Mamba, Transformer, CNN+Transformer, GCN+Transformer, LSTM+Transformer. LSTM+Mamba is the core component LSTMS6 of the EchoMamba framework proposed by us. In order to ensure the consistency and reliability when applied to different models, the data sets are preprocessed by RSFM strategy before the model is used to process the data sets.

By combining CNN, GCN, and LSTM with Mamba and Transformer, respectively, the experimental design aims to systematically verify how different feature extraction paradigms such as local perception, graph structure modeling, and temporal memory complement or compete with the two types of core architectures (SSM and Attention).

Table 3 provides a detailed comparison of the average performance of these two benchmark models across ten independent repeated experiments on the validation sets of six different hyperspectral image datasets. In order to analyze the performance of the model from as comprehensive a perspective as possible, while retaining the traditional performance indicators such as overall accuracy (OA), and Kappa coefficient (κ), we also introduced the key performance indicator of model training time, which is measured in min. Which defined as the time it took to train the model over 200 cycles. Through this comprehensive evaluation method, this paper aims to deeply analyze how the model can effectively reduce the training time while maintaining high classification performance when processing HSI data.

**Table 3:** Performance comparison of validationsets.

DataSet		Model							
		Mamba benchmark model				Transformer benchmark model			
		Null	CNN	GCN	LSTM (EchoMamba)	Null	CNN	GCN	LSTM
Ausburg	Train time(min)	0.6477	0.7276	0.9365	0.7768	10	11	9	12
	OA	1.0000	0.9945	0.9862	1.0000	0.9823	0.9943	0.9341	1.0000
	κ	1.0000	0.9911	0.9886	1.0000	0.9744	0.9917	0.9134	1.0000
Salinas	Train time(min)	88	96	77	99	1045	1112	803	1147
	OA	0.9667	0.9432	0.9636	0.9799	0.9811	0.9803	0.9782	0.9795
	κ	0.9676	0.9621	0.9601	0.9813	0.9823	0.9754	0.9732	0.9788
Pavia Centre scene	Train time(min)	195	220	224	226	3548	3741	3529	3892
	OA	0.9244	0.9321	0.9280	0.9567	0.9423	0.9688	0.9732	0.9522
	κ	0.8899	0.9071	0.8992	0.9367	0.9266	0.9602	0.9588	0.9332
Pavia University	Train time(min)	52	56	69	61	969	1041	777	1059
	OA	0.9571	0.9622	0.9777	0.9978	0.9821	0.9911	0.9903	0.9869
	κ	0.9431	0.9478	0.9732	0.9930	0.9822	0.9923	0.9929	0.9828
Indian pines	Train time(min)	12	15	16	15	218	227	245	231
	OA	0.9786	0.9634	0.9870	0.9951	0.9973	0.9967	0.9922	0.9926
	κ	0.9777	0.9621	0.9844	0.9931	0.9965	0.9943	0.9956	0.9942
Houston 2013	Train time(min)	6	6	10	7	56	105	98	106
	OA	0.9822	0.9534	0.9889	0.9895	0.9913	0.9909	0.9959	0.9907
	κ	0.9796	0.9488	0.9832	0.9877	0.9904	0.9889	0.9957	0.9901

The Transformer benchmark model has shown extremely excellent performance on the three HSI data sets, and the OA is maintained at a high level. However, in terms of training time, the Transformer benchmark model generally takes a long time for data training, which requires a high time cost.

The Mamba benchmark model demonstrated excellent performance on six separate HSI datasets. In particular, on the Salinas dataset, all four Mamba benchmark models achieved an overall accuracy of at or near 100% on the validation set with very short training times. Compared with Mamba, CNN+Mamba achieved certain performance improvement in the processing of two datasets, Pavia Centre scene and Pavia University, but did not show obvious advantages in the other four datasets. GCN+Mamba has demonstrated superior performance compared to CNN+Mamba on datasets such as Pavia University, Indian Pines, Houston, etc. In some aspects, this model even surpasses the performance of EchoMamba. Although the six datasets used have some differences in data characteristics, from the performance analysis, compared with the other three Mamba benchmark models, the EchoMamba model has achieved improvements ranging from 0.01% to 4.99% in each performance metric with only slightly increased training time.

As can be seen from the Train time, with the same training period set, the time cost required by the Mamba benchmark model to train the data set is much smaller than that of the Transformer benchmark model. In addition, in the two indexes of OA and κ, Mamba benchmark model and Transformer benchmark model showed very similar performance on data set processing, and even showed better performance than Transformer benchmark model on some data sets. This result shows that compared with the Transformer benchmark model, the Mamba benchmark model can achieve very close performance while greatly improving the training efficiency.

Due to the comprehensive advantages of the Mamba benchmark model compared to the Transformer benchmark model, we will not consider using the Transformer benchmark model during the in-depth comparison of the model’s experimental performance on the test set of the dataset in the following section. Instead, we will focus on a more comprehensive and in-depth performance comparison of the Mamba benchmark model. Through this comparison, it is expected to provide a more solid and reliable experimental basis for model selection and optimization.

### Test set performance comparison

In order to comprehensively evaluate the performance of Mamba variant models, we introduce a more comprehensive set of evaluation indicators in the processing of test set. In addition to using the population Accuracy (OA) as the core performance indicator, we also calculated the average accuracy (AA) to ensure careful consideration of the recognition accuracy of each class of samples in the HSI. In addition, to provide an in-depth analysis of the model’s performance, we introduced the F1 score, which balances accuracy and recall to give a more complete picture of the model’s classification. We recorded the performance of each framework in ten independent repeated experiments on different datasets in the form of standard deviation. Additionally, we added confidence intervals to the OA index to measure the differences in performance stability among different models. Table 4 shows the performance of the Mamba benchmark model in predicting the test set of six HSI datasets.

**Table 4:** Performance comparison of test set.

DataSet		Index				
		OA	OA confidence interval	AA	κ	F1
Ausburg	Mamba	0.9940 ± 0.0058	[0.9882, 1.0000]	0.9932 ± 0.0063	0.9928 ± 0.0053	0.9223 ± 0.0055
	CNN+Mamba	0.9481 ± 0.0052	[0.9383, 0.9579]	0.9402 ± 0.0053	0.9300 ± 0.0049	0.9523 ± 0.0059
	GCN+Mamba	0.9932 ± 0.0053	[0.9892, 1.0000]	0.9939 ± 0.0054	0.9942 ± 0.0047	0.9933 ± 0.0052
	EchoMamba (LSTM+Mamba)	0.9970 ± 0.0030	[0.9941, 1.0000]	0.9965 ± 0.0033	0.9970 ± 0.0023	0.9966 ± 0.0034
Salinas	Mamba	0.9655 ± 0.0033	[0.9586, 0.9724]	0.9842 ± 0.0036	0.9617 ± 0.0038	0.9848 ± 0.0041
	CNN+Mamba	0.9472 ± 0.0038	[0.9394, 0.9550]	0.9766 ± 0.0042	0.9413 ± 0.0046	0.9770 ± 0.0044
	GCN+Mamba	0.9601 ± 0.0032	[0.9542, 0.9660]	0.9828 ± 0.0036	0.9555 ± 0.0031	0.9830 ± 0.0037
	EchoMamba (LSTM+Mamba)	0.9865 ± 0.0021	[0.9826, 0.9904]	0.9941 ± 0.0025	0.9850 ± 0.0022	0.9941 ± 0.0029
Pavia Centre scene	Mamba	0.9242 ± 0.0022	[0.9203, 0.9281]	0.8250 ± 0.0023	0.8943 ± 0.0027	0.8546 ± 0.0026
	CNN+Mamba	0.9343 ± 0.0022	[0.9299, 0.9387]	0.8401 ± 0.0027	0.9080 ± 0.0022	0.8690 ± 0.0022
	GCN+Mamba	0.9316 ± 0.0018	[0.9281, 0.9351]	0.9136 ± 0.0023	0.9039 ± 0.0013	0.8606 ± 0.0017
	EchoMamba (LSTM+Mamba)	0.9544 ± 0.0010	[0.9525, 0.9563]	0.8750 ± 0.0015	0.9363 ± 0.0009	0.9100 ± 0.0008
Pavia University	Mamba	0.9600 ± 0.0040	[0.9522, 0.9678]	0.9381 ± 0.0045	0.9490 ± 0.0043	0.9473 ± 0.0041
	CNN+Mamba	0.9537 ± 0.0045	[0.9449, 0.9625]	0.9322 ± 0.0051	0.9405 ± 0.0047	0.9393 ± 0.0046
	GCN+Mamba	0.9776 ± 0.0037	[0.9707, 0.9845]	0.9788 ± 0.0042	0.9705 ± 0.0036	0.9725 ± 0.0037
	EchoMamba (LSTM+Mamba)	0.9972 ± 0.0027	[0.9923, 1.0000]	0.9960 ± 0.0032	0.9962 ± 0.0027	0.9965 ± 0.0023
Indian Pines	Mamba	0.9814 ± 0.0031	[0.9755, 0.9873]	0.9852 ± 0.0036	0.9867 ± 0.0031	0.9787 ± 0.0034
	CNN+Mamba	0.9561 ± 0.0037	[0.9492, 0.9630]	0.9692 ± 0.0040	0.9712 ± 0.0034	0.9496 ± 0.0034
	GCN+Mamba	0.9873 ± 0.0021	[0.9824, 0.9922]	0.9918 ± 0.0031	0.9856 ± 0.0023	0.9908 ± 0.0026
	EchoMamba (LSTM+Mamba)	0.9903 ± 0.0015	[0.9874, 0.9932]	0.9916 ± 0.0021	0.9888 ± 0.0013	0.9924 ± 0.0017
Houston 2013	Mamba	0.9781 ± 0.0022	[0.9732, 0.9830]	0.9793 ± 0.0032	0.9787 ± 0.0025	0.9762 ± 0.0022
	CNN+Mamba	0.9668 ± 0.0031	[0.9609, 0.9727]	0.9707 ± 0.0039	0.9702 ± 0.0036	0.9641 ± 0.0031
	GCN+Mamba	0.9934 ± 0.0023	[0.9895, 0.9973]	0.9948 ± 0.0027	0.9928 ± 0.0021	0.9936 ± 0.0023
	EchoMamba (LSTM+Mamba)	0.9840 ± 0.0012	[0.9816, 0.9864]	0.9840 ± 0.0016	0.9847 ± 0.0012	0.9827 ± 0.0014

Based on the results of the standard deviation and the confidence interval of OA, it can be seen that the performance of each group of models is within a relatively stable range. In theory, CNN+Mamba, as a variant of Mamba, should demonstrate performance beyond the original Mamba model. However, in practical tests, for the five datasets of Augsburg, Pavia Centre scene, Pavia University, Indian Pines and Houston 2013, the predictive performance of CNN+Mamba model is slightly lower than that of the original Mamba model. In the performance comparison of the verification set, GCN+Mamba and CNN+Mamba have their own advantages and disadvantages in the processing performance of the six data sets, and do not show obvious advantages. However, in the performance comparison of the test set, compared with the performance of CNN+Mamba, GCN+Mamba has achieved all-round breakthroughs. It even shows better performance than EchoMamba in the processing of the Houston 2013 dataset. But overall, the EchoMamba model is significantly better than the other three models in the prediction performance indexes for all the test set of data used, showing excellent performance. The results show that CNN+Mamba does not achieve a comprehensive improvement in performance compared with Mamba. GCN+Mamba outperformed CNN+Mamba in results, but the overall performance was still not comparable to EchoMamba. However, EchoMamba model shows better prediction ability than GCN+Mamba, CNN+Mamba and Mamba.

**Fig 5:**
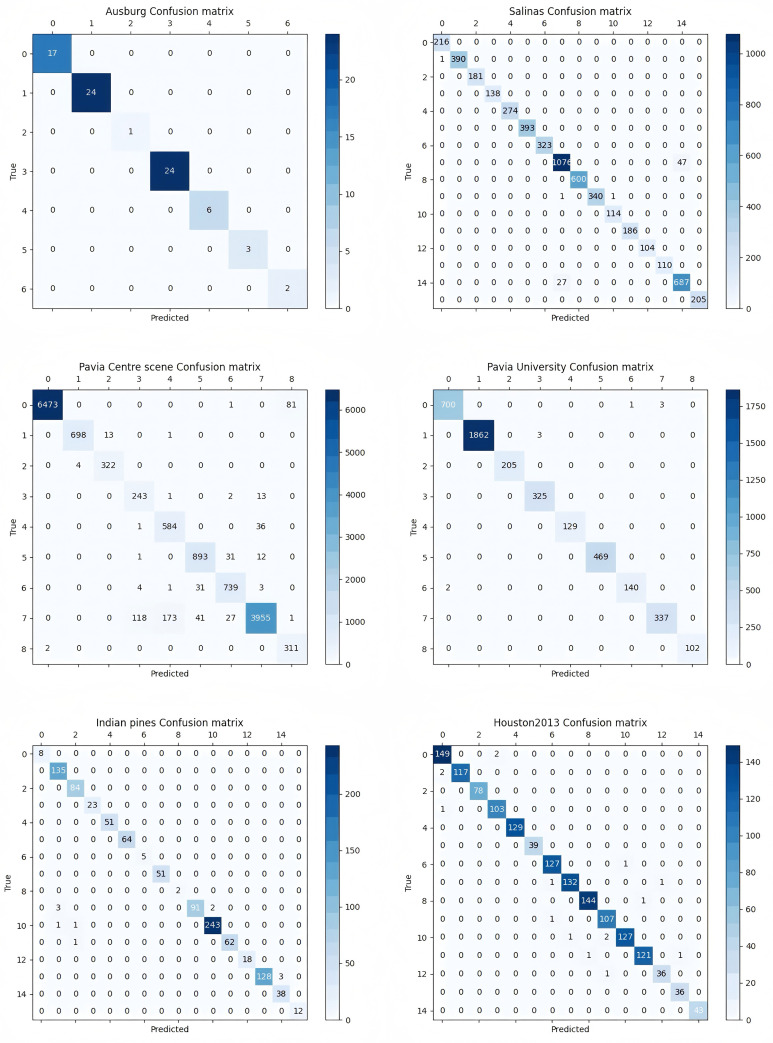
Test set confusion matrix with EchoMamba.

To further confirm the superiority of EchoMamba model, Fig 5 shows the prediction confusion matrix of EchoMamba model on the test set of six data sets(Let’s take one of the ten independent repeated experiments as an example). These confusion matrices clearly show the classification accuracy of the model across different categories, thus further validating the superiority of the EchoMamba model in the HSI classification task.

The analysis of the confusion matrix revealed some challenges that EchoMamba still faced in its classification performance on the evaluated datasets. On the Ausburg dataset, there were obvious misclassification phenomena between the category 3 and category 5, as well as between the category 6 and category 7. Similarly, on the Salinas dataset, the misclassification rate between the category 8 and category 10 was relatively high. On the Pavia University dataset, the performance was good, and no intermittent high misclassification rates for specific categories were observed; it is notable that EchoMamba achieved a perfect 100% accuracy rate for the category 7. On the Houston 2013 dataset, the classification accuracy for the category 9 was extremely high (98.4%), but its main misclassification was caused by confusion with the category 7.

To specifically evaluate the robustness of EchoMamba in the presence of imbalanced sample distribution, we focused on the Indian Pines dataset and the Pavia Centre scene dataset, both of which have significant class imbalance issues. For the Indian Pines dataset, its sample distribution was extremely unbalanced, and EchoMamba made many errors when classifying the categories with very few samples (specifically categories 1 and 16). The results on the Pavia Centre scene dataset further highlighted the influence of sample quantity: for the categories with a larger number of samples, the classification accuracy reached 99.2%, but for the categories with a smaller number of samples, the accuracy dropped significantly to 89.7%. This phenomenon indicates that when dealing with datasets with uneven sample distribution, there is still much room for improvement in EchoMamba’s generalization ability.

To sum up, EchoMamba is compared with several advanced models in terms of overall performance. These models include Mamba, CNN+Mamba, GCN+Mamba, Transformer, CNN+Transformer, GCN+Transformer, LSTM+Transformer. Through rigorous experimental testing and evaluation, we draw the following conclusions: in the process of processing HSI data sets, EchoMamba has shown more powerful model performance than other benchmark models in terms of feature extraction, classification accuracy, and model generalization ability.

In the second stage of the experiment, we will continue to explore the EchoMamba model and make a detailed performance comparison between the EchoMamba model and the improved Mamba model proposed by other researchers mentioned earlier in the field of hyperspectral image processing. Through this series of comparisons, we hope to further verify the superiority of EchoMamba model and contribute new insights to the development of HSI processing technology.

### Advanced experiment

In this stage of the experiment, we compare the performance of EchoMamba with the more advanced variant model 3DSS-Mamba mentioned earlier to explore the advantages and disadvantages of both. We selected three datasets, Pavia University, Indian Pine and Houston 2013, which completely coincided with the research of He et al [24]. to conduct a detailed test on the performance of 3DSS-Mamba. In order to ensure the consistency of experimental conditions, we strictly followed the partitioning method of data sets proposed by He et al.: For the Pavia University dataset, we adopted the partitioning ratio of 5% training set and 95% test set; And for Indian Pines and Houston 2013 data sets, 10% training set and 90% test set were divided.

We used the complete EchoMamba framework, which includes RFMS data preprocessing strategies and LSTMS6 model components, to sequentially train and predict three reclassified data sets, and compared the performance of 3DSS-Mamba.

In order to further verify the performance of EchoMamba, we replicated another advanced framework – the 3D-CNN HSI classification framework [34]. We compared its performance with that of EchoMamba by placing it alongside 3DSS-Mamba as a competing model. In Table 5, we provide a detailed comparative analysis of the classification prediction performance of EchoMamba, 3DSS-Mamba and 3D-CNN on three datasets covering accuracy, overall accuracy (OA), average accuracy (AA), and Kappa coefficient (κ) for each category of features.

**Table 5:** Performance comparison between 3DSS-Mamba and EchoMamba.

Class	Pavia University			Indian Pines			Houston 2013		
	EchoMamba	3DSS-Mamba	3D-CNN	EchoMamba	3DSS-Mamba	3D-CNN	EchoMamba	3DSS-Mamba	3D-CNN
1	0.9811	0.9933	0.9922	1.0000	0.7805	0.9202	0.9992	0.9902	0.9887
2	0.9955	0.9934	0.9821	0.9951	0.9027	0.9129	0.9960	0.9956	0.9647
3	0.9938	0.9509	0.9611	0.9916	0.9304	0.9455	1.0000	1.0000	0.9276
4	0.9967	0.9591	0.9550	1.0000	0.9343	0.9445	0.9976	0.9768	0.9821
5	0.9993	0.9969	0.9928	0.9876	0.9770	0.9236	0.9992	0.9991	0.9964
6	0.9980	0.9943	0.9933	1.0000	0.9726	0.9828	1.0000	0.9829	0.9987
7	1.0000	0.9588	0.9721	1.0000	0.9600	0.9543	0.9976	0.9755	0.9653
8	0.9902	0.9620	0.9533	1.0000	0.9930	0.9882	1.0000	0.9616	0.9789
9	1.0000	0.9722	0.9678	1.0000	0.3889	0.6709	0.9840	0.9627	0.9553
10				0.9990	0.9680	0.9338	0.9976	0.9955	0.9931
11				0.9939	0.9891	0.9762	0.9984	0.9820	0.9632
12				1.0000	0.9139	0.8991	0.9984	0.9838	0.9281
13				1.0000	0.9568	0.9567	0.9893	0.9668	0.9765
14				0.9573	0.9965	0.9768	1.0000	0.9948	0.9932
15				0.9845	0.9452	0.9543	1.0000	1.0000	0.9765
16				1.0000	0.8452	0.8932			
OA	0.9935	0.9848	0.9801	0.9906	0.9582	0.9568	0.9970	0.9837	0.9725
AA	0.9865	0.9756	0.9712	0.9896	0.9083	0.9271	0.9971	0.9844	0.9726
κ	0.9914	0.9798	0.9754	0.9893	0.9523	0.9501	0.9968	0.9824	0.9727

The results show that EchoMamba outperforms 3DSS-Mamba and 3D-CNN on all test datasets. In particular, on some categories, 3DSS-Mamba and 3D-CNN failed to achieve 90% accuracy, while EchoMamba was able to achieve almost 100% prediction accuracy. This significant difference fully reflects the advanced nature and practical value of EchoMamba in the field of HSI processing. In addition, visually demonstrate EchoMamba’s predictive effect on these three data sets, further confirming its superior performance.

**Fig 6:**
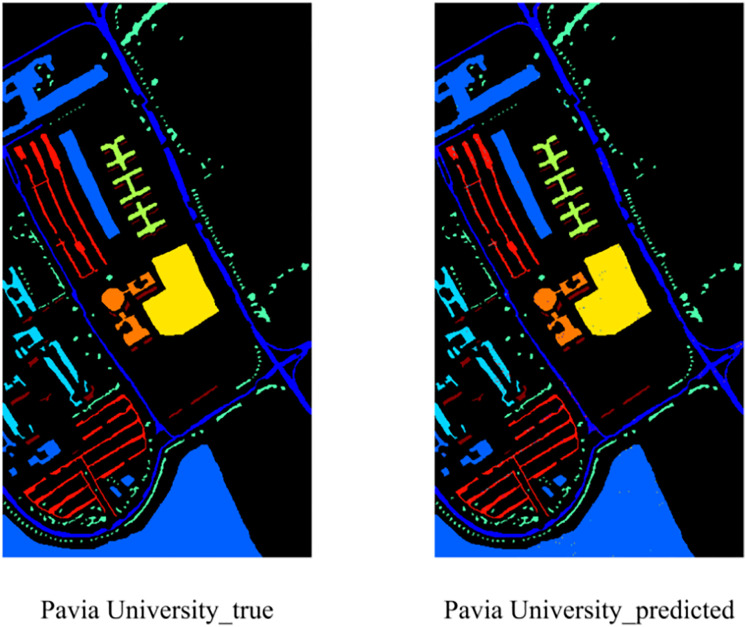
The predictive effect of EchoMamba on Pavia University.

**Fig 7:**
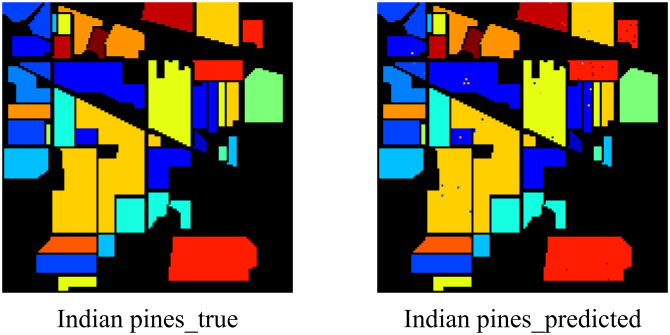
The predictive effect of EchoMamba on Indian Pines.

**Fig 8:**
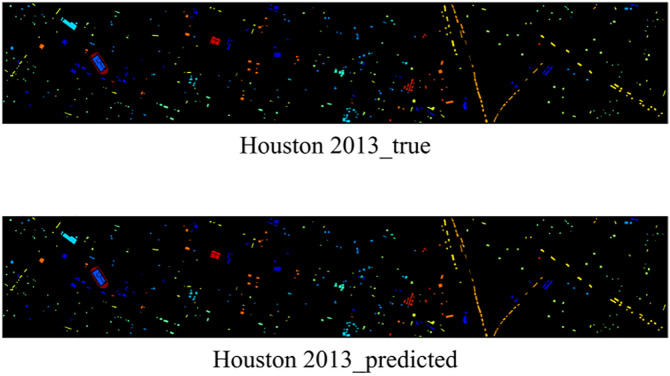
The predictive effect of EchoMamba on Houston 2013.

### Parameter adjustment

On the basis of following the data set partitioning principle in advanced experiment, we dig deeply into the performance of EchoMamba model. In order to maximize the performance of the model, we carried out fine parameter adjustment and optimization.

First of all, in the core component of EchoMamba, LSTMS6, we use the unidirectional LSTM module by default, although the combination of unidirectional LSTM and Mamba can achieve better performance than the combination of CNN, GCN and Mamba. However, to ensure maximum use of LSTM in EchoMamba, we took a closer look at the original LSTM module and compared the performance of unidirectional LSTM, bidirectional LSTM and GRU in combination with Mamba, respectively as shown in Table 6.

**Table 6:** Further performance comparison of LSTM modules.

DataSet	Model(+Mamba)	OA	AA	κ
Pavia University	Unidirectional LSTM	0.9979	0.9974	0.9972
	Bidirectional LSTM	0.9979	0.9970	0.9971
	GRU	0.9955	0.9936	0.9941
Indian Pines	Unidirectional LSTM	0.9951	0.9919	0.9944
	Bidirectional LSTM	0.9844	0.9851	0.9822
	GRU	0.9951	0.9874	0.9944
Houston 2013	Unidirectional LSTM	0.9840	0.9835	0.9829
	Bidirectional LSTM	0.9840	0.9839	0.9827
	GRU	0.9842	0.9851	0.9835

In the processing of the three data sets, Unidirectional LSTM as a module is concatenated on the Mamba model to achieve the most powerful performance, although Bidirectional LSTM+Mamba can theoretically better learn HSI data correlation. However, the performance of Unidirectional LSTM+Mamba is not exceeded. Therefore, according to Table 6, the combination of Unidirectional LSTM and Mamba can better realize the role of LSTM layer in EchoMamba framework.

In LSTMS6, the core component of the EchoMamba model, the location of the LSTM layer and its dimensional scale are two key factors. In the experiments in Chapter 2, we placed the LSTM layer above the S6 layer by default. In order to investigate the effect of the LSTM layer position on the performance of the EchoMamba model in depth, the following attempts were made: The LSTM layer was placed in the first layer of LSTMS6 (i.e., the layer above the S6 model) and the last layer (i.e., the layer below the S6 model). We trained these two models on three datasets: Pavia University, Indian Pines, and Houston 2013. After testing, the performance of the two models on the test set is shown in Table 7.

**Table 7:** Performance comparison of LSTM layers at different locations in LSTMS6.

DataSet	Model	OA	AA	κ
Pavia University	EchoMamba1 (Upper LSTM)	0.9978	0.9961	0.9970
	EchoMamba2 (Lower LSTM)	0.7848	0.6580	0.7229
Indian Pines	EchoMamba1 (Upper LSTM)	0.9922	0.9927	0.9911
	EchoMamba2 (Lower LSTM)	0.4488	0.5978	0.3964
Houston 2013	EchoMamba1 (Upper LSTM)	0.9971	0.9969	0.9968
	EchoMamba2 (Lower LSTM)	0.7117	0.7102	0.6885

As shown in Table 7, EchoMamba1 has significantly better performance than EchoMamba2 in various verification indicators. This finding indicates that placing the LSTM layer in the first layer of LSTMS6, in other words, placing the LSTM layer on top of the S6 layer, can significantly improve the performance of the EchoMamba model and achieve optimal performance.

**Fig 9:**
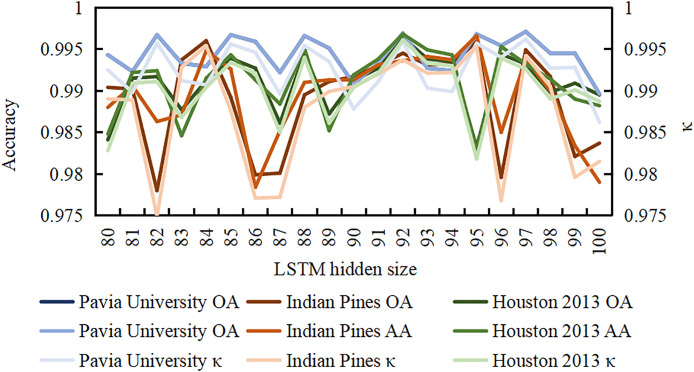
Adjustments for LSTM hidden layer dimensions.

In order to solve the problem of dimension scale of LSTM layer, we set the dimension of LSTM to 90 in Chapter 2, and the data set is processed to achieve a better result. In order to further explore whether EchoMamba can achieve better performance in HSI data processing, we modified the dimension of LSTM layer, and tried it successively from 80 to 100 around the original set of 90. The changing trend of model performance is shown in Fig 9.

As shown in Fig 9, for OA, AA, κ and other indicators of the three datasets, when LSTM’s hidden layer dimension is 92, EchoMamba’s OA, AA, and κ at Pavia University are 0.9969, 0.9947, and 0.9959. On Indian Pines, OA was 0.9945, AA was 0.9936, κ was 0.9936; Houston 2013 has OA of 0.9964, AA of 0.9968, κ of 0.9961, which can achieve the best model performance across the three datasets.

In adjusting the learning rate of the model, we conducted a careful experimental comparison, testing four different learning rate Settings of 0.1, 0.01, 0.001 and 0.0001 respectively. After observation and analysis of a series of training cycles, we found that when the learning rate was set to 0.1 and 0.01, the model showed a certain volatility in the training process. Specifically, in the early training period, the performance index of the model gradually increased, showing a good learning effect. However, in the middle and late training period. However, the performance of the model under the learning rate setting suddenly and rapidly decreased, and the phenomenon of gradient explosion appeared; When the learning rate is set to 0.0001 and 0.00001, the performance of the model is gradually improved in the training stage, but the performance on the test is far worse than the performance on the training, which may be due to the overfitting of the model. Different from the first two cases, when the learning rate is set to 0.001, the model shows a more robust learning process. With the increase of training cycle, the performance index of the model continues to rise, and finally stabilizes at a higher level, and also shows excellent performance on the test set. By comparing the experimental results, we come to the conclusion that 0.001 is the most suitable learning rate for EchoMamba among the tested learning rates, which enables the model to achieve the best performance in the training process, so as to learn the training set features of HSI data more thoroughly and apply them to the test set.

To sum up, we achieve the optimal performance of EchoMamba through parameter adjustment. When the LSTM layer is placed in the first layer of LSTMS6 and the dimension is set to 92, the learning rate of the model is set to 0.01, which can play the best prediction and classification ability of EchoMamba on HSI.

## Discussion

In basic experiment, we have observed a significant phenomenon: although the Mamba benchmark model has a significant reduction in training time compared with the Transformer benchmark model, its predictive performance is very similar to that of the Transformer benchmark model or even better in some data sets. HSI datasets have a large amount of data and complex dimensions. Each pixel contains numerous spectral bands, each band corresponds to a time step, resulting in extremely large data series length. Therefore, in the training process of HSI, training time cost has become an important index to measure model performance. The Mamba benchmark model, with its linear time complexity, shows a significant training speed advantage when processing such long sequences of HSI data. In addition, the built-in selectivity mechanism of the Mamba model allows the model to selectively focus on or ignore certain information according to the characteristics of the input data, which not only effectively compresses the state space, but also greatly improves the processing efficiency of the model. Redundant information and noise are common problems in HSI data. Mamba’s own selectivity mechanism can effectively screen out useful information and filter out interference factors, thus further improving its performance. In contrast, the Transformer benchmark model has a unique self-attention mechanism to capture complex relationships in the data, but this also means a lot of matrix multiplication and nonlinear calculation in the process, which is not only computationally heavy, but also the memory consumption of the device is extremely challenging. Therefore, compared with Mamba benchmark model, Transformer benchmark model has obvious shortcomings in terms of processing speed.

In the internal comparison of the Mamba benchmark model, it can be observed that compared with the original Mamba model, the performance of CNN+Mamba integrated into the CNN layer has not been significantly improved. On the contrary, the performance of CNN+Mamba is even worse than that of Mamba in some datasets. This phenomenon likely stems from the combination of CNN and Mamba increases the complexity of the model, resulting in some overfitting problems in the model, which makes it difficult for CNN+Mamba to generalize the learning of the training set to the test set and even the whole data set. Due to the dimensional complexity of HSI, the spectral information may be sufficient for the task of image prediction and classification. The additional spatial information learned by combining CNN may not bring further performance improvement of the model, and even increase the noise of the model. With its selective mechanism, Mamba pays more attention to the spectral dimension information of HSI data, while CNN pays more attention to the learning of spatial dimension information. In addition, in the CNN+Mamba model, information fusion is also a major problem, and improper fusion strategies may also lead to data loss and confusion, thus reducing the performance of the model.

When processing HSI, GCN+Mamba shows better performance than CNN+Mamba, which may be mainly due to their complementary adaptation to HSI data characteristics. HSI data has the characteristics of high-dimensional continuous spectral features coupled with local spatial structure, and its pixels form a long sequence correlation of non-Euclidean manifolds in spectral dimension, while there are local similarities in spatial dimension. GCN models the global relationship between pixels or regions through the graph structure, regards each pixel as a graph node, uses spectral similarity or spatial adjacency to build edge weights, and can explicitly capture long-distance dependence and non-local feature interaction in high-dimensional space. This capability is especially suitable for the continuity and redundancy characteristics of HSI spectral dimensions. However, the convolution kernel of traditional CNN is limited by the assumption of local sensitivity field and invariance of displacement, so it is difficult to model the global nonlinear relationship in spectral dimension, and the low spatial resolution of HSI may introduce noise in the extraction of spatial features. As a state-space model, Mamba can model long sequences efficiently through a selective state mechanism. When the graph structure features extracted by GCN are input, the implied state can further optimize the spectrum-space joint representation, dynamically screen important band information along the sequence dimension and suppress redundancy. However, if the front-end uses CNN, the regular grid features of its output may have lost part of the global spectral association information, which makes it difficult for Mamba to fully exploit the complex cross-band dependence. In addition, the graph convolution operation of GCN realizes frequency domain filtering through Laplacian matrix, which is physically consistent with the spectral signal processing requirements of HSI data in essence, while the spatial convolution operation of CNN is more inclined to spatial texture extraction. The difference of inductive bias between the two in HSI tasks may eventually lead to the combination of GCN+Mamba being more effective in collaborative modeling of the deep semantic information of spectral-spatial dual modes.

EchoMamba stands out in the performance of four Mamba benchmark models. This may be due to the multi-scale dynamic modeling of spectrum-space complex dependence through the deep collaboration mechanism between LSTM and Mamba. The spectral sequence of HSI data not only contains long distance global associations, but also local timing patterns and dynamic context sensitivity. LSTM can explicitly capture the local timing dependence and short-term memory of spectral dimensions with its gated loop structure, and filter noise and redundancy adaptively through gradient-controlled information flow, while preserving the context coherence of key spectral features. Mamba, on the other hand, uses a selective state space model for efficient global modeling of long sequences, and its dynamic update mechanism of hidden states can focus on cross-band strongly correlated features and suppress irrelevant interference. The combination of the two forms a double enhancement of “local segmentation - global abstraction” during feature transfer: LSTM first extracts local temporal features and compacts memory of the original spectral sequence to alleviate the information overload problem that may be caused by the direct input of long sequences into Mamba, and at the same time retains the local spectral response mode sensitive to classification through the gating mechanism. Mamba then further explores the global nonlinear relationship in spectral dimension and the implicit cross-band interaction based on the higher-order temporal characterization of LSTM output, and its selectivity mechanism can dynamically adjust the weight of attention to the features of different time steps in LSTM memory units, thus achieving a more detailed hierarchical representation at the spectrum-space joint embedding level. In addition, the cyclic iterative characteristics of LSTM are complementary to the parallel long series processing capability of Mamba. The former enhances the sensitivity of the model to subtle spectral differences through gradual iteration, while the latter enhances the generalization ability of the overall distribution law through efficient global modeling. This structural design may be more suitable for the classification requirements driven by local spectral features and global distribution patterns in HSI data.

The improvement idea of 3DSS-Mamba represents the mainstream way of Mamba in the field of HSI data processing, that is, HSI is regarded as a 3D data cube, and spectral spatial features are extracted by convolutional operation, and feature selection and information propagation are carried out by Mamba model to realize the fusion of spectral and spatial information. This method effectively realizes the combination of spatial dimension and spectral dimension. However, like the CNN+Mamba benchmark model we used, 3DSS-Mamba has a relatively complex design of model structure in order to comprehensively learn multidimensional data features, while EchoMamba regards HSI bands as time series and uses LSTM to capture long-term dependence between bands. And combined with the feature extraction capability of Mamba model to realize the effective utilization of spectral features. This approach appropriately reduces the model’s attention to the HSI spatial dimension, on the contrary, it focuses on the in-depth mining of the spectral dimension, and the model structure is relatively simple. Compared with 3DSS-Mamba in advanced experiment performance comparison, EchoMamba shows a comprehensive advantage, which proves the practicability and advanced nature of this framework.

In the study of EchoMamba’s LSTM module, we tried to replace it with bidirectional LSTM, GRU. However, the results show that the processing performance of unidirectional LSTM combined with Mamba on three common data sets is stronger than that of bidirectional LSTM and GRU as module concatenation.Hyperspectral data has significant local spatial-spectral correlation, and its classification task is highly dependent on local context information of pixel neighborhood or band, and unidirectional LSTM one-way information flow exactly conforms to this local causality -- through the dynamic regulation of forgetting gate and input gate, It accurately captures key features of the preceding band or adjacent pixels while avoiding interference from non-causal noise. Although bidirectional LSTM can theoretically merge the global context, in HSI scenarios, the “future information” introduced by the reverse sequence may destroy the physical coherence of local features, causing the model to confuse the core discriminant features. Especially when the data size is limited, the complex parameter number of bidirectional structure is more likely to cause overfitting, further weakening performance. Although the GRU reduces the computation cost by simplifying the gating mechanism, its coarse-grained control of information flow is inadequate in processing HSI high-dimensional nonlinear spectral features, and it is difficult to accurately screen and transmit multi-level features through independent gating like LSTM.

During the adjustment of EchoMamba, we observed that the position of LSTM in LSTMS6 plays an important role in the performance of the model. When LSTM is placed in the first layer of the model, that is, the upper layer of S6, the performance of EchoMamba can be achieved far better than that of LSTM placed in the last layer. This may be because LSTM can directly learn the local features and timing information of the image at the input layer, and use it as the input of S6, so as to help S6 better capture the context information and long-distance dependence relationship of the image, realize the close combination of feature extraction and timing modeling, and improve the richness of feature representation. In addition, LSTM can directly integrate the learned time sequence information into the feature extraction process of S6 at the input layer to achieve efficient fusion of information transfer and avoid additional integration and modeling at the output layer, thus simplifying the model structure, reducing the computational complexity, and improving the reasoning speed and output quality of the model.

While EchoMamba has demonstrated significant advantages in HSI classification tasks, it still faces limitations and challenges that require further exploration and improvement. EchoMamba primarily focuses on spectral feature extraction, with limited exploitation of spatial information. When EchoMamba flattens the spatial data of HSI, the change of dimension may destroy the spatial feature relationship of the original HSI. This may lead to performance degradation when dealing with tasks sensitive to spatial information, such as high-resolution HSI image classification or object detection. To further enhance the performance and applicability of EchoMamba, future research directions can be concentrated on integrating spatial feature extraction into the model.

## Conclusions

This paper proposes an HSI data processing model based on Mamba, EchoMamba, which combines a series of data preprocessing strategies with LSTM and Mamba model, and utilizes LSTM ability to capture the relationship between bands and Mamba’s powerful feature extraction ability. By sacrificing some spatial relationship learning capability, we can conduct more in-depth exploration of the deep spectral relationships within HSI data. A large number of experiments have proved that the EchoMamba proposed in this paper and the HSI classification model based on Transformer, as well as some traditional Mamba improved models that integrate spatial features with spectral features, have more powerful classification and prediction ability. This study provides a new feasible solution for the HSI classification task. In the future, the focus of the research will be on exploring the scalability of the Mamba model in a wider range of hyperspectral scenarios.
